# The influence of acoustic startle probes on fear learning in humans

**DOI:** 10.1038/s41598-018-32646-1

**Published:** 2018-09-28

**Authors:** Michelle I. C. de Haan, Sonja van Well, Renée M. Visser, H. Steven Scholte, Guido A. van Wingen, Merel Kindt

**Affiliations:** 1 Amsterdam Brain and Cognition (ABC), University of Amsterdam, Amsterdam, The Netherlands; 2 Department of Psychiatry, Amsterdam Neuroscience, Amsterdam UMC, University of Amsterdam, Amsterdam, The Netherlands; 3 Department of Clinical Psychology, University of Amsterdam, Amsterdam, The Netherlands; 4 Medical Research Council, Cognition and Brain Sciences Unit, University of Cambridge, Cambridge, United Kingdom

## Abstract

Even though human fear-conditioning involves affective learning as well as expectancy learning, most studies assess only one of the two distinct processes. Commonly used read-outs of associative fear learning are the fear-potentiated startle reflex (FPS), pupil dilation and US-expectancy ratings. FPS is thought to reflect the affective aspect of fear learning, while pupil dilation reflects a general arousal response. However, in order to measure FPS, aversively loud acoustic probes are presented during conditioning, which might in itself exert an effect on fear learning. Here we tested the effect of startle probes on fear learning by comparing brain activation (fMRI), pupil dilation and US-expectancy ratings with and without acoustic startle probes within subjects. Regardless of startle probes, fear conditioning resulted in enhanced dACC, insula and ventral striatum activation. Interaction analyses showed that startle probes diminished differential pupil dilation between CS+ and CS− due to increased pupil responses to CS−. A trend significant interaction effect was observed for US-expectancy and amygdala activation. Startle probes affect differential fear learning by impeding safety learning, as measured with pupil dilation, a read-out of the cognitive component of fear learning. However, we observed no significant effect of acoustic startle probes on other measures of fear learning.

## Introduction

While fear conditioning involves both affective and cognitive learning, few studies actually include read-outs of both cognitive and affective learning. In affective learning the aversive valence of the unconditioned stimulus (US) is transferred to the conditioned stimulus (CS), while in expectancy learning the CS becomes a predictor of the US on a cognitive level^1,2^. According to the dual process theory of fear learning, separate systems are involved in the formation and expression of the emotional and cognitive aspects of fear learning and these processes can occur independent of each other^1,3–8^. In order to disentangle the underlying brain mechanisms of these different read-outs of fear, multiple measures of fear learning within a functional magnetic resonance imaging (fMRI) study are required, given that emotional and cognitive learning are deemed to be reflected in dissociating correlates. Evidence of fear learning in humans is usually obtained through behavioral and physiological measures of the expression of fear, such as the fear-potentiated startle (FPS) reflex, pupil dilation, skin conductance response and US-expectancy ratings and fMRI^9^. FPS is the augmentation of the eye blink component of the startle reflex that occurs during fear conditioning. The amygdala increases the amplitude of the eye blink reflex during fear conditioning through three parallel (direct and indirect) pathways involving the central nucleus of the amygdala, the pontis caudalis, the superior colliculus, the deep mesencephalic reticular nucleus, the medial nucleus of the amygdala, the ventromedial hypothalamus and the periaqueductal gray^10^. FPS differs from pupil dilation and skin conductance response in that it is a specific measure of fear that, unlike skin conductance and pupil dilation, differentiates between positive and negative valence and occurs independent of the awareness of CS−US contingencies^4,11–13^. In contrast to FPS, fear-conditioning as measured with skin conductance seems to mirror US-expectancy ratings^7,8,14^. Together these findings indicate that FPS reflects the affective aspects of fear learning, while pupil dilation and skin conductance reflect expectancy learning and general arousal^5,8,10,15,16^. Currently FPS is the preferred measure when studying the emotional aspects of fear learning, even though the FPS does not always differentiate between positive and negative affect^17,18^. However, measurement of FPS requires an additional experimental manipulation. Loud acoustic startle probes are presented to elicit a startle reflex, which are themselves also aversive^19^. Hence, administering additional aversive stimuli during fear conditioning (in a fMRI context) might affect the formation of fear associations, as indexed by physiological and neural read-outs. Indeed, in a between group paradigm, Sjouwerman and colleagues found that differential skin conductance responding was attenuated by administration of acoustic startle probes (driven by decreased responding to CS+)^20^. Here, we examined the influence of startle probes on both behavioral and neural read-outs of fear learning, by simultaneously assessing brain activation (fMRI), affective learning (FPS) and expectancy learning (retrospective US-expectancy ratings and pupil dilation) during fear conditioning.

Previous fear-conditioning fMRI studies in humans show activation of a large network of brain regions (what we will refer to as the ‘fear-network’) composed of anterior insular cortex, dorsal anterior cingulate cortex (dACC), thalamus, pre-supplementary and supplementary motor cortex, ventral striatum, dorsal-anterior precuneus, second somatosensory cortex, dorsolateral prefrontal cortex, lateral premotor cortex, ventral-posterior precuneus, lateral cerebellum, septal-hypothalamic zone, midbrain/dorsal pons and pontomedullary junction^21^. To our knowledge, so far only two studies have assessed concurrent fMRI and fear-potentiated startle as indices of fear learning^22,23^. Both studies combined measurement of fear-potentiated startle with fMRI during a fear-conditioning task and showed typical fear-network activation. However, they did not specifically test whether startle probes had an influence on different aspects of fear learning. Unlike most fear conditioning fMRI studies^21^, amygdala activation was in addition to fear-network activations observed in both studies^22,23^. Therefore, we predicted that acoustic startle probes might enhance fear learning (in the amygdala), as indexed by a stronger differentiation between CS+ and CS−. In the current study, we assessed expectancy learning by use of US-expectancy ratings and pupil dilation. We used pupil dilation instead of the more frequently used skin conductance response, because pupil dilation data is relatively easy to acquire, and the conditioning effect on skin conductance is often less strong due to a relatively high level of noise and large within- and between-subject variability^15,24,25^. To our knowledge, the effect of acoustic startle probes on fear learning as measured with pupil dilation has not been studied before. Animal studies show that pupil dilation is a relatively direct measure of locus coeruleus (LC) activation^26^. Unexpected acoustic beeps elicit LC activity^27^, which leads to pupil dilation^28^. In humans, pupil dilation response has been shown to occur in anticipation of the US during fear conditioning^15,29–32^. Pupil dilation is also thought to reflect an orienting response^33,34^ and a continued state of arousal has been found to increase tonic pupil diameter thereby decreasing phasic pupil responses^35^. In addition to the LC, pupil dilation responses during fear learning have been found to correlate with activation in the dACC and to a lesser degree to insula and thalamus^15^. These regions are part of the fear-network, or more generally, the salience-network^21,36,37^. It remains however elusive whether acoustic startle probes actually affect the fear-conditioned changes in pupil dilation and brain activity in the fear-network.

**Figure 1:**
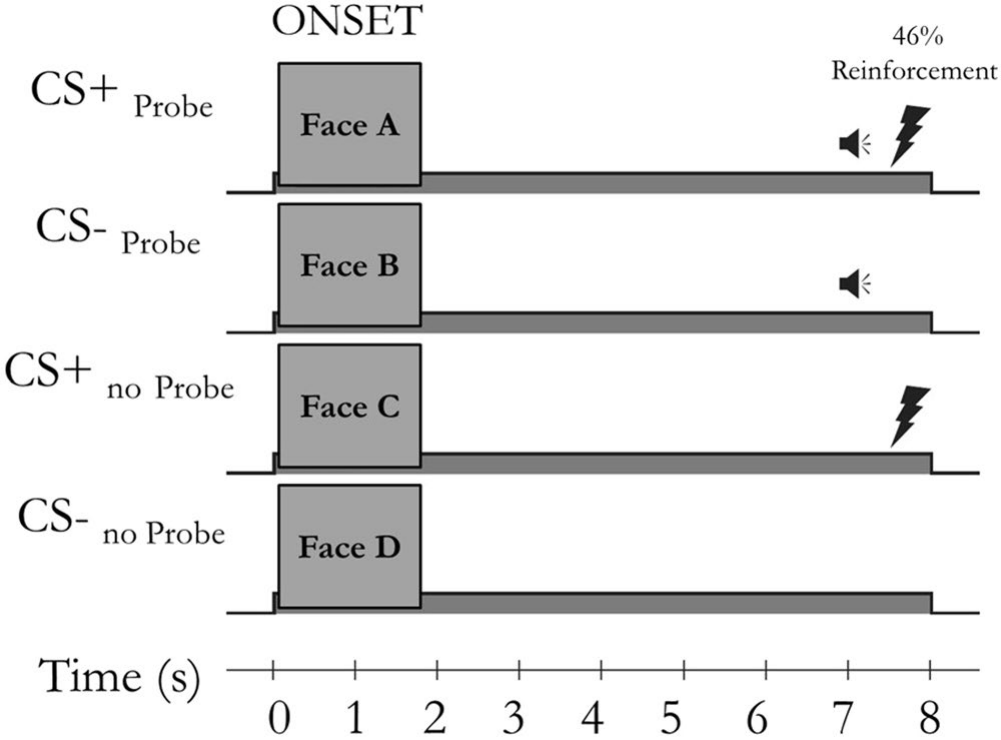
Task design showing the four within-subject conditions (CS+_Probe_, CS−_Probe_, CS+_No probe_ and CS−_No probe_) and the timing of the acoustic startle probe and US onsets. Throughout fear acquisition, each CS was presented 13 times, with 46% of the CS+ presentations being reinforced with the US.

To address this question fear conditioning with and without startle probes was compared within subject while simultaneously assessing brain activity. We assessed the effect of acoustic startle probes on brain activation (both whole brain and in specific regions of interest (ROIs); the dACC, insula, ventral striatum, thalamus, midbrain/dorsal pons and amygdala separately) and pupil dilation during fear conditioning and on retrospective US-expectancy ratings. As CSs we used pictures of neutral faces with two separate categories (i.e., age: adult vs. child and sex: male vs. female) both categories consisted of a CS+ and a CS− stimulus, but only one category was paired with acoustic startle probes (Fig. 1).

## Methods

### Participants

Twenty-six healthy volunteers (20 male, 6 female, all right handed) aged between 19 and 42 years (*M* = 25.2, *SD* = 6.8) participated in this experiment. All participants were right-handed, reported no psychiatric or neurological disorders, no current use of any psychoactive medication and no previous experience with a fear-conditioning procedure. The ethical committee of the University of Amsterdam approved the study protocol (2012-KP-2346) and all procedures were carried out in accordance with the guidelines and regulations. All participants signed informed consent and received financial compensation for their participation.

### Experimental design

The experimental design consisted of two conditions: fear conditioning with and without acoustic startle probes (Fig. 1). Four different pictures served as a to-be conditioned stimulus (CS), two of which were paired with an acoustic startle probe on every presentation (CS_Probe_) and the other two were never paired with a startle probe (CS_No probe_). One of the CS_Probe_ stimuli (CS+_Probe_) was, in addition to the startle probe, also paired with the unconditioned stimulus (US) and the other was never paired with the US (CS−_Probe_). Likewise, of the two CS_No probe_ stimuli, one was paired with the US (CS+_No probe_) and the other was never paired with the US (CS−_No probe_).

### Stimuli and conditioning procedure

The experiment consisted of one session of fMRI scanning during which we used a classical fear-conditioning paradigm, with delay conditioning and partial reinforcement. Prior to scanning, anxiety sensitivity and state anxiety were assessed with the Anxiety Sensitivity Index (ASI; Peterson and Reiss, 1992) and the State Anxiety inventory (STAI-S; Spielberger, 1983) respectively.

An electrical shock served as the US and was delivered twice for 2 ms, with a delay of 300 ms^30,32^, by a Digitimer DS7A constant-current stimulator (Hertfordshire, U.K.) through MRI-compatible carbon electrodes attached to the participant’s right wrist. The intensity of the electrical shock was individually adapted by manually triggering the electrical stimuli while the participant was laying on the scanner table. We started at an intensity of 1 mA and continued administering single shocks with a gradual but non-linear increase in intensity (1, 2, 4, 6, 8, 11, 15, 18, 21, 24, 28, 32 mA etc.) until the participant indicated that the electrical stimulus was “clearly uncomfortable, difficult to tolerate, but not painful” (intensity range 8–70 mA, *M* = 25, *SD* = 14.8). The other two stimuli were never reinforced (CS−_Probe_ and CS−_No probe_).

Four pictures of neutral faces served as CSs. The images (obtained from Shutterstock, Inc.) were selected according to age (adult vs. child) and sex (male vs. female). All faces were cropped, scaled to equal height, and separated from their background using Adobe Photoshop software (CS5). Images were then placed on a gray background and adjusted on mean brightness. The two CSs+, as well as the two CSs−, were opposites (i.e., male adult and female child, or female adult and male child, etc.) in order to counteract generalization on basis of sex or age. Assignment of the images as CS+, CS− and Probe, No probe was counterbalanced across participants. Visual stimuli were backward-projected onto a screen that was viewed through a mirror attached to the head coil. Participants were further instructed to look carefully at the images, as some of the images would be paired with the electrical shock. Two pictures co-occurred with an acoustic startle probe (100 dB, 40 ms burst of broadband noise administered binaurally through headphones) on every presentation (CSs_Probe_) and the other two were never paired with the probe (CSs_No probe_). We used a MRconfon audiosystem (Cambridge Research Systems Ltd, Rochester, Kent, UK) without active noise-cancelling to administer the acoustic startle probes. Participants did not wear earplugs, instead they were protected from the scanner noise by extra foam cushions, including cushions that tightly fit around the headphones, sealing off the scanner noise.

To reduce initial startle reactivity and to familiarize participants with the pictures, fear acquisition was preceded by a habituation phase consisting of six acoustic startle probes presented alone (i.e., noise alone, (NA; interval: 12, 15 or 18 s, *M* = 15 s) and a pre-exposure phase consisting of the first three presentations of each of the four to-be CSs (CS+_Probe_, CS−_Probe_, CS+_No probe_ and CS−_No probe_) and the NA. During the pre-exposure phase, acoustic startle probes, but not yet the USs were administered. Then, throughout fear acquisition, each CS was presented 13 times, with 46% of the CS+ presentations being reinforced with the US. Again, acoustic startle probes were administered on CS_Probe_ trials, but not CS_No Probe_ trials. In addition, 13 NA acoustic startle probes were presented to obtain baseline startle responses.

The duration of CS presentation was 8 s in total. During the 8 s CS presentations the acoustic startle probe was presented 7 s after CS onset (on CS+_Probe_ and CS−_Probe_ trials) and the US was presented 7.5 s after CS onset (on CS+_Probe_ and CS+_No probe_ trials; Fig. 1). The relatively long interval between CS onset and acoustic startle probe and US was used to minimize their influence on the neuroimaging and pupil dilation data. The inter-trial interval (ITI) varied between 12, 15, and 18 s (*M* = 15 s). Trial order and ITIs were semi-random, such that no more than two consecutive trials or ITIs were of the same type. After scanning, awareness of the CS−US contingency was first assessed verbally. Thereafter, participants gave written retrospective US-expectancy ratings, evaluated the US and startle probe and filled in the second STAI-S and a Trait Anxiety Inventory (STAI-S and STAI-T; Spielberger, 1983).

## Measures

### Self-report questionnaires

The Anxiety Sensitivity Index (ASI; Peterson & Reiss, 1992) was used to assess participants’ tendency to respond fearfully to anxiety-related symptoms. Their degrees of state and trait anxiety were assessed using the State–Trait Anxiety Inventory (STAI; Spielberger, Gorsuch, & Lushene, 1970).

### Fear-potentiated startle reflex

The conditioned fear response was measured as potentiation of the acoustic startle reflex. The eye blink component of the reflex was acquired through electromyography (EMG) measurements of the right orbicularis oculi muscle^38^. We used disposable MR compatible carbon electrodes (Kendall H135TSG 35 mm) with a vinyl tape backing. Part of the adhesive strip of the electrode (approximately 20 mm) was cut off to allow for convenient positioning under the participants eye. To prevent possible warming of the electrodes in the scanner we used carbon electrode leads with carbon current-limiting resistors serially connected between the lead and electrode^39^. Electrode leads were twisted to minimize gradient artefacts on the EMG recordings and connected to an MRI-compatible amplifier (Geodesic EEG system 300 MR, EGI, USA) that was placed outside the scanner bore and grounded through a RF filter. Data were sampled at 1000 Hz and recorded using NetStation software (version 4.5.2, Electrical Geodesics, Inc. (EGI), Eugene, USA).

Unfortunately we could not analyze the fear-potentiated startle data because the quality of the signal that remained after offline filtering (to remove MRI artefacts) was insufficient. We tried to offline correct the raw EMG signal for scanner artefacts. Multiple different filter options from NetStation software and Brain Vision Analyzer software (version 1.05, Brain Products GmbH, Munich, Germany) were applied. Scanner artefacts were removed by subtracting an artefact template from the EMG data using a sliding average of 2, 5, 10, 15, 20 and 25 consecutive volumes. In addition, we removed the scanner artefact with all other available options in NetStation; by applying Gaussian removal, averaging over all volumes, and calculating an exponential average with a 0.5, 0.10 or 0.15 smoothing factor. Every filter successfully removed the MRI-artefact from the EMG data, however none was able to distinguish between FPS signal and MRI-artefact and therefore the FPS signal was removed from the data together with the MRI-artefact and only a low frequency (<28 Hz) signal component of the FPS signal remained, which likely reflects a motion artefact^38^.

### Pupil dilation

Pupil dilation responses were recorded continuously using a non-ferromagnetic eye tracker with fiber optic camera upgrade (EyeLink 1000, SR Research Ltd., Mississauga, Ontario, Canada). Data were sampled at 250 Hz. Subsequently, pupil dilation responses were calculated as the peak in pupil diameter during stimulus presentation (i.e., 0–6.5 s after stimulus onset) from baseline (i.e., 500 ms prior to stimulus onset). Data samples that were obscured by eye blinks (more than 10 ms of missing consecutive samples) and the 100 ms before and after each blink were discarded and replaced by a linear trend at point, using the entire time series. Furthermore, trials containing substantial signal loss, affecting more than 50% of the baseline or the 6.5 s after stimulus onset, were regarded as unreliable and replaced by estimating the linear trend at point over trials for each condition separately (*M* = 3.1%, *SD* = 4.2%).The applied methods were similar to previous studies from our group^30–32^. Finally, pupil dilation responses were converted to t-scores within participants.

### Neuroimaging

Imaging was conducted using a 3 T MRI scanner (Philips, Achieva XT) with a 32-channel head coil. Whole-brain functional MRI images were acquired: GE-EPI, TR = 2000 ms, TE = 27.6 ms, FA = 76.1°, FOV = 240 mm; matrix = 80 × 80; slice thickness = 3 mm; 37 axial slices sequentially acquired). Additionally, a T1-weighted anatomical image was obtained for each participant (TR = 8.2 ms; TE = 3.7 ms; FA = 8°; FOV = 240 × 188 mm; matrix = 240 × 240; slice thickness = 1 mm; 220 axial slices sequentially acquired).

### Retrospective US-expectancy and aversion ratings of US and acoustic startle probe

Participants retrospectively rated the likelihood of US delivery for each stimulus type for the beginning (pre-exposure), mid (early acquisition), and end (late acquisition) of the fear conditioning task on an 11-point Likert scale (−5: certainly not, 0: maybe, and 5: certainly). In addition, the US and acoustic startle probe were evaluated for averseness by means of the arousal and valence dimensions of the Self-Assessment Manikin^40^. Participants rated the stimuli on the pictorial dimensions of arousal and valence on a 9-point Likert scale (1: excited and positive, 9: calm and negative).

## Analysis

### Pupil dilation and retrospective US-expectancy ratings

Pre-exposure (trials 1–3) was compared to late acquisition (trials 10–16). Accordingly, pupil dilation responses and US-expectancy ratings were separately computed for the pre-exposure and late acquisition phase. To statistically examine the effect of acoustic startle probes on fear learning (i.e., pupil dilation, US-expectancy ratings), we used repeated measures ANOVAs (SPSS, version 23) with Stimulus (CS+ vs. CS−), Trial (pre-exposure vs. late acquisition), and Condition (Probe vs. No probe) as within-subjects variables and with a significance level of *p* < 0.05. Where appropriate, Greenhouse-Geisser corrections were applied to control for the violation of the sphericity assumption. In addition, arousal and valence ratings of startle probes and US were analyzed using paired samples *t*-tests (two-tailed). Post-hoc *t*-tests were tested one-tailed with a significance level of *p* < 0.05.

### Neuroimaging

Imaging data were processed and analyzed using FSL (FMRIB’s Software Library) software. First, functional images were motion corrected (MCFLIRT)^41^, spatially smoothed (5 mm full-width-at-half-maximum Gaussian kernel), and high-pass filtered (cutoff = 100 s). Structural images were brain extracted (BED)^42^. Subsequently, for each participant, functional images were aligned to the structural image and transformed, on the basis of this structural image, to standard space (MNI) using a 7-degree-of-freedom affine registration followed by linear warping.

Thereafter, functional MRI data were analyzed using general linear models. Stimulus onsets for the four conditions (CS+_Probe_, CS−_Probe_, CS+_No Probe_ and CS−_No Probe_) were modelled as double Gamma functions with a duration of 0 ms, leaving a time window of 7 s until onset of the startle probe in the Probe condition and 7.5 s until the onset of the US in the reinforced CS+ trials. Furthermore, the USs and acoustic startle probes (as well as the motion parameters in six directions and the temporal derivatives of the task-regressors) were included in the model as regressors of no interest to ensure estimation of the variance uniquely explained by the CSs. Contrasts for the four conditions versus baseline were specified on the first single-subject level. Then, on the second single-subject level, the main effect of Stimulus (CS+/CS−) across the Probe and No probe condition (CS+_Probe_ and CS+_No Probe_ > CS−_Probe_ and CS−_No Probe_), the main effect of Condition (Probe/No probe) across CS+ and CS− (CS+_Probe_ and CS−_Probe_ < > CS+_No Probe_ and CS−_No Probe_), and the interaction between Stimulus and Condition were specified.

Voxel-wise statistical tests were family-wise error rate corrected for multiple comparisons (*p* < 0.05) for the whole brain or the ROIs using threshold free cluster enhancement (TFCE)^43^ with 5000 permutations. The six ROIs were defined on the basis of a recent meta-analysis of fear-conditioning studies^21^ and included the dACC, insula, ventral striatum, thalamus and midbrain/dorsal pons. In addition, we included the amygdala because of its hypothesized involvement in fear-potentiated startle^5,10^. The subcortical regions (ventral striatum, amygdala and thalamus) were anatomically defined using the Harvard-Oxford subcortical structural atlas as implemented in FSL (probability ≥25%). The cortical regions (dACC and insula) and midbrain/dorsal pons were created centered around peak activation coordinates obtained from the literature. We used 15-mm spheres for dACC left/right: x = 8/−10, y = 18/6, z = 42/44 and for insula left/right: x = −40/40, y = 18/16, z = −2/2^21^. The midbrain/dorsal pons was defined as two box masks (10 × 10 × 22 mm) centered around the periaqueductal grey (PAG) x = 0, y = −30, z = −12^44^ and the LC x = 2, y = −38, z = −28^45^. All coordinates are reported in MNI standard space. We performed additional ROI analyses to obtain non-biased effect sizes. Therefore, we extracted the mean z-value on single subject level from the individual ROIs (FSL Featquery) and conducted repeated measures ANOVA (SPSS, version 23) to calculate partial eta-squared. For both types of fMRI analyses, we used Bonferroni correction for six ROIs, resulting in an adjusted significance level of 0.0083.

The datasets generated and analysed during the current study are available in the Open Science Framework repository; https://osf.io/e8vbn/.

## Results

### Study sample

Data from several participants were excluded entirely from the analyses due to excessive sleepiness as monitored with pupil recordings (n = 5) and technical difficulties with administration of the acoustic probes (n = 2). One participant was excluded from the MRI and pupil dilation analysis due to incomplete data transportation (n = 1). Two others were excluded only from pupil dilation analysis because over 25% of the trials within one condition were obscured by eye blinks (n = 2). Thus, the remaining samples on which analyses were performed consisted of 16 participants for pupil dilation and 18 participants for MRI and US-expectancy ratings. For ASI, STAI-S and STAI-T scores see Table 1.

**Table 1:** Self-reported state and trait anxiety and anxiety sensitivity scores for the participants included in the fMRI analysis (n = 18).

	Mean	Standard deviation	Minimum score	Maximum score
ASI	7.78	5.00	2	23
STAI-S pre-scan	32.22	7.86	20	47
STAI-S post-scan	33.89	6.85	20	45
STAI-T	35.78	9.66	23	57

### Aversion ratings US and startle probe

We assessed whether the US and the startle probe differed on valence and arousal reported by participants. We found no significant difference (*t*(17) <1) between the level of arousal elicited by the US (*M* = 4.83, *SD* = 2.20) and the startle probe (*M* = 5.39, *SD* = 1.88). Likewise, we found no significant difference (*t*(17) <1) between the valence reported for the US (*M* = 6, *SD* = 1.85) and the startle probe (*M* = 6.5, *SD* = 1.2), suggesting that the US and startle probe were equally aversive.

### US-expectancy ratings

**Figure 2:**
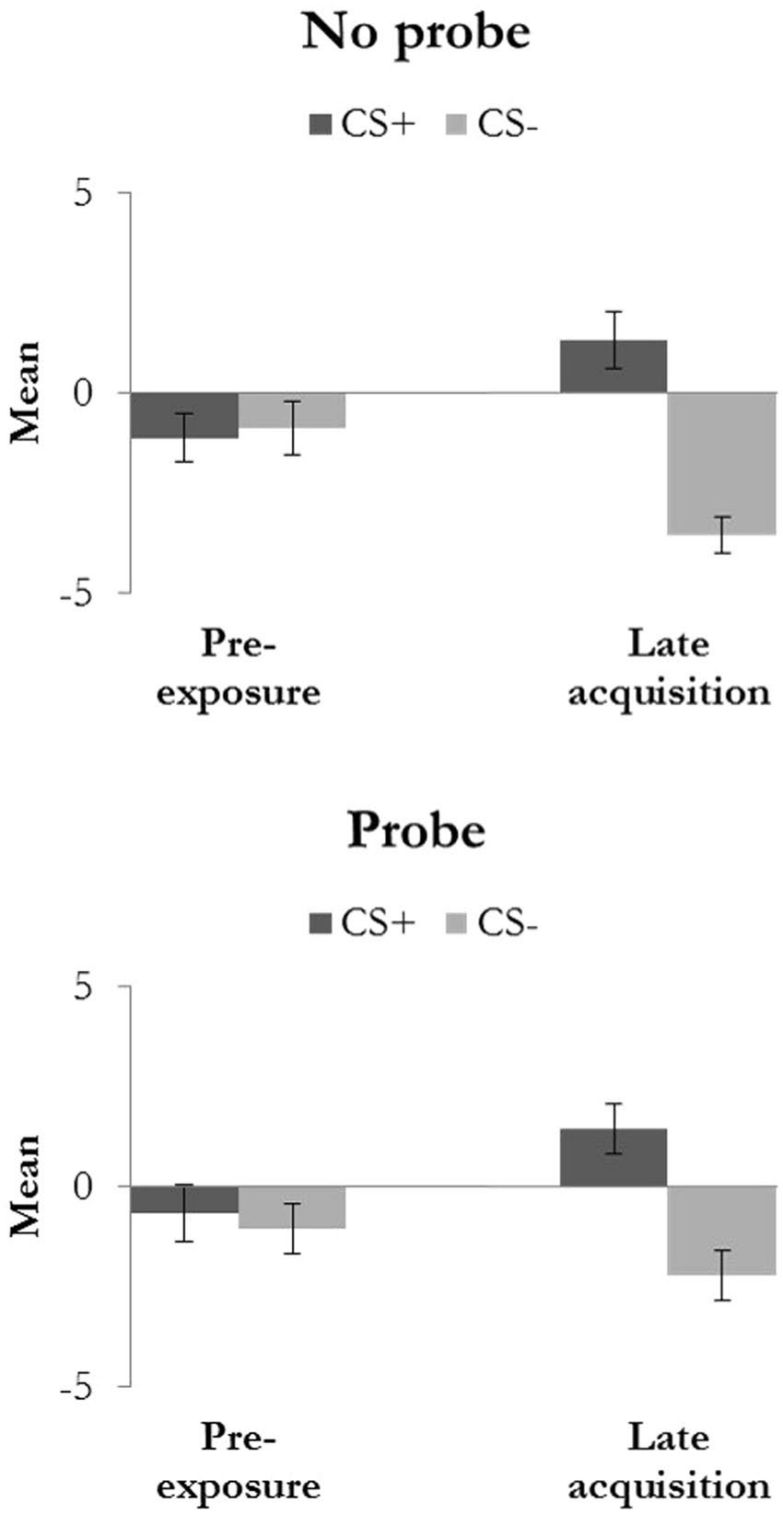
Retrospective US-expectancy ratings for the pre-exposure and late acquisition for the Probe and No probe condition. Error bars represent standard error of the mean.

We compared retrospective US-expectancy ratings (CS+ vs. CS−) during pre-exposure to late acquisition and analyzed the effect of startle probes on expectancies. Analyses of variance showed marginally significant less differentiation of US-expectancies in the Probe compared to the No probe condition (Stimulus × Trial × Condition: *F(*1,17) = 4.04, *p* = 0.06, η_p_^2^ = 0.19; Fig. 2). The main effect of Stimulus was significant *F(*1,17) = 25.94, *p* < 0.001, η_p_^2^ = 0.60) and the main effects of Trial (*F(*1,17) = 0.08, *p* = 0.78, η_p_^2^ = 0.01) and Condition (*F(*1,17) = 1.22, *p* = 0.28, η_p_^2^ = 0.07) were not significant. The interaction effect between Stimulus and Trial was significant (*F(*1,17) = 16.72, *p* = 0.001, η_p_^2^ = 0.50) and the interaction effects between Stimulus and Condition (*F(*1,17) = 0.38, *p* = 0.55, η_p_^2^ = 0.02) and between Trial and Condition (*F(*1,17) = 0.65, *p* = 0.43, η_p_^2^ = 0.04) were not significant.

### Pupil dilation

**Figure 3:**
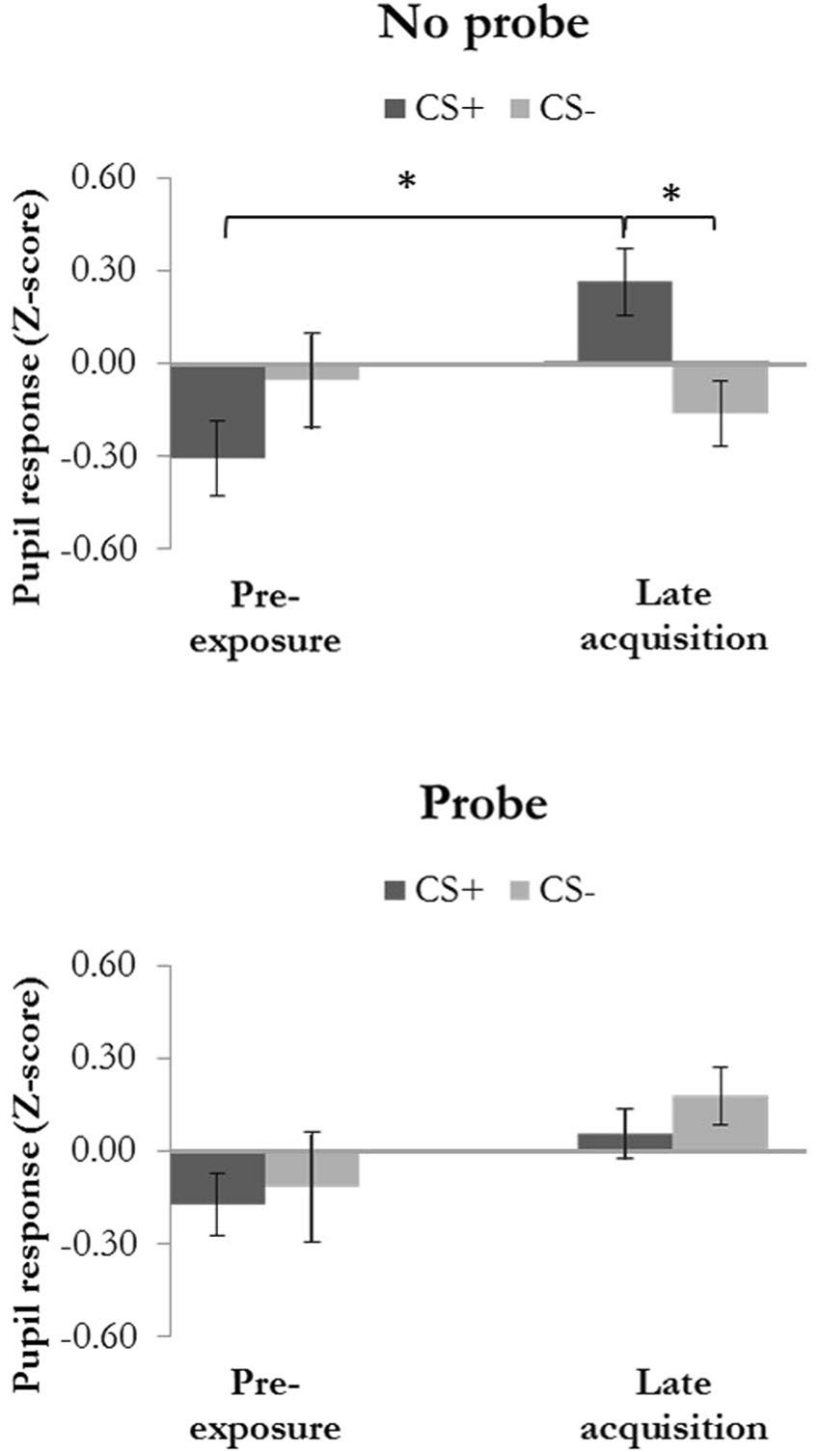
Pupil dilation response (Z-scores) during pre-exposure and late acquisition for the Probe and No probe condition. Error bars represent standard error of the mean. *Indicate significant differences (post-hoc t-tests tested one-tailed).

We compared differential pupil dilation (CS+ vs. CS−) during pre-exposure to late acquisition and assessed the effect of startle probes on conditioning (Fig. 3). If more than 25% of the trials within one condition had to be discarded, the participant was excluded from pupil dilation analysis.

Analyses of variance showed that fear learning, as indexed by pupil dilation, differed in the Probe as compared to the No probe condition (Stimulus × Trial × Condition: *F(*1,15) = 5.29, *p* < 0.05, η_p_^2^ = 0.26). The main effect of Stimulus (*F(*1,15) = 0.00, *p* = 0.99, η_p_^2^ = 0.00) and the main effect of Condition (*F(*1,15) = 0.92, *p* = 0.35, η_p_^2^ = 0.06) were not significant, while the main effect of Trial was significant (*F(*1,15) = 5.20, *p* = 0.04, η_p_^2^ = 0.26). The interaction effects between Stimulus and Trial (*F(*1,15) = 2.55, *p* = 0.13, η_p_^2^ = 0.15), Stimulus and Condition (*F(*1,15) = 0.95, *p* = 0.35, η_p_^2^ = 0.06) and Trial and Condition (*F(*1,15) = 0.03, *p* = 0.87, η_p_^2^ = 0.00) were not significant.

The No probe condition showed successful fear learning, indicated by a significant increase in differential pupil dilation (CS+ vs. CS−) from pre-exposure to late acquisition (Stimulus × Trial: *F(*1,15) = 8.65, *p* = 0.01, η_p_^2^ = 0.37), whereas the Probe condition showed no fear acquisition, as indicated by a lack of differentiation between the CS+ and CS− in the probe condition (Stimulus × Trial: *F(*1,15) <1.0).

That is, in the No probe condition only, the fear-conditioned stimulus (CS+) elicited significantly larger pupil dilation responses than did the control stimulus (CS−) during late acquisition (*t*(15) = 2.22, *p* < 0.05, one-tailed, *d* = 0.56). To confirm, CS+ and CS− did not differ during pre-exposure(*t*(15) <1.0). To better understand the lack of a significant interaction effect in the Probe condition, we performed an additional exploratory analysis, testing whether the lack of a difference was due to a lower-than-usual CS+ response or an elevated CS− response. As it turned out, the lack of differential pupil dilation (CS+ vs. CS−) in the Probe condition was driven by elevated pupil dilation to the CS− as compared to the No Probe condition (*t*(15) = 2.58, *p* = 0.01, one-tailed, *d* = 0.64) during late acquisition, but not during pre-exposure (*t*(15) <1.0).

In addition, we assessed whether a difference in number of gaze shift artefacts could have influenced our findings. With a repeated measures ANOVA we tested whether the number of saccades differed between conditions. We found a trend significant main effect of stimulus (*F(*1,15) = 3.72, *p* = 0.07, η_p_^2^ = 0.20), no significant main effect of condition (*F(*1,15) = 0.02, *p* = 0.89, η_p_^2^ = 0.00) and no significant interaction between stimulus and condition (*F(*1,15) = 0.00, *p* = 1.00, η_p_^2^ = 0.00). Hence, the decreased differentiation in the Probe condition cannot be explained by a difference in number of saccades.

## Neuroimaging

**Figure 4:**
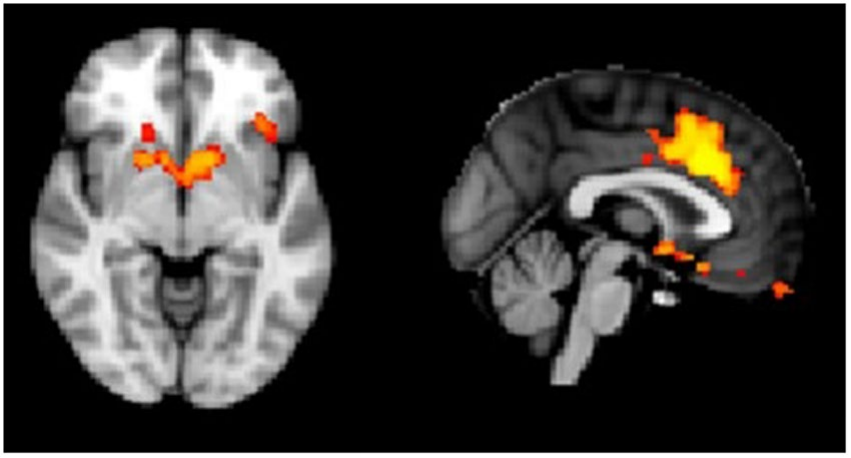
Activation for the main effect of conditioning, irrespective of startle probes (contrast CS+ > CS−, p_TFCE_ <0.05).

The main effect of Stimulus across the Probe and No probe conditions (CS+ > CS−) showed significant conditioning in the insula, dACC, lateral occipital cortex, planum temporale, frontal pole, superior temporal gyrus, occipital pole, superior frontal gyrus, precentral gyrus, angular gyrus, middle frontal gyrus, posterior supramarginal gyrus and superior frontal gyrus on whole brain level (Fig. 4) and also in nucleus accumbens after small volume correction (Table 2). Moreover, the main effects CS−> CS+ and Probe >/< No Probe, did not show significant voxels.

**Table 2:** Peak coordinates of significantly activated brain regions. We applied threshold free cluster enhancement (TFCE). *Significant on whole brain level with an α of 5%.

Contrast	Region	Side	MNI- coördinates			*p*-value	*t*-score
			X	Y	Z		
CS+ > CS−	OFC	L	52	78	25	*p* = 0.004*	6.2
	Insula	L	62	77	35	*p* = 0.019*	5.0
	dACC		45	72	51	*p* < 0.001*	7.1
	Cuneal cortex	L	51	20	58	*p* = 0.027*	4.8
			67	20	38	*p* = 0.029*	5.2
	Planum temporale	L	75	49	43	*p* = 0.039*	4.0
	Frontal pole	L	65	85	51	*p* = 0.036*	5.1
	Superior temporal gyrus	L	77	63	36	*p* = 0.041*	4.4
	Occipital pole	L	63	16	42	*p* = 0.036*	5.3
	Superior frontal gyrus	L	52	67	71	*p* = 0.036*	5.4
	Precentral gyrus	L	70	65	60	*p* = 0.046*	4.4
	Angular gyrus	L	63	36	54	*p* = 0.045*	4.5
	Precentral gyrus	L	65	65	65	*p* = 0.049*	4.1
	Posterior supramarginal gyrus	L	62	40	54	*p* = 0.048*	4.3
	Superior frontal gyrus	L	52	71	70	*p* = 0.047*	4.9
	Accumbens	L	49	68	30	*p* < 0.001**	4.5
		R	42	66	32	*p* = 0.003**	4.3
Interaction CS x Probe	Amygdala	L	52	62	25	*p* = 0.029***	3.7

**Significant after small volume correction and after Bonferroni correction of α for the number of ROIs (six ROIs). ***Significant after small volume correction, but not after Bonferroni correction of α for the number of ROIs (six ROIs). The contrasts CS− > CS+, Probe > No probe and No probe > Probe yielded no significantly activated voxels.

The Stimulus × Condition interaction showed an effect of startle probes on fear conditioning in the amygdala (t(17) = 3.7, *p* = 0.029). However, this effect did not reach the threshold of significance after Bonferroni correction of α for the number of ROIs.

**Figure 5:**
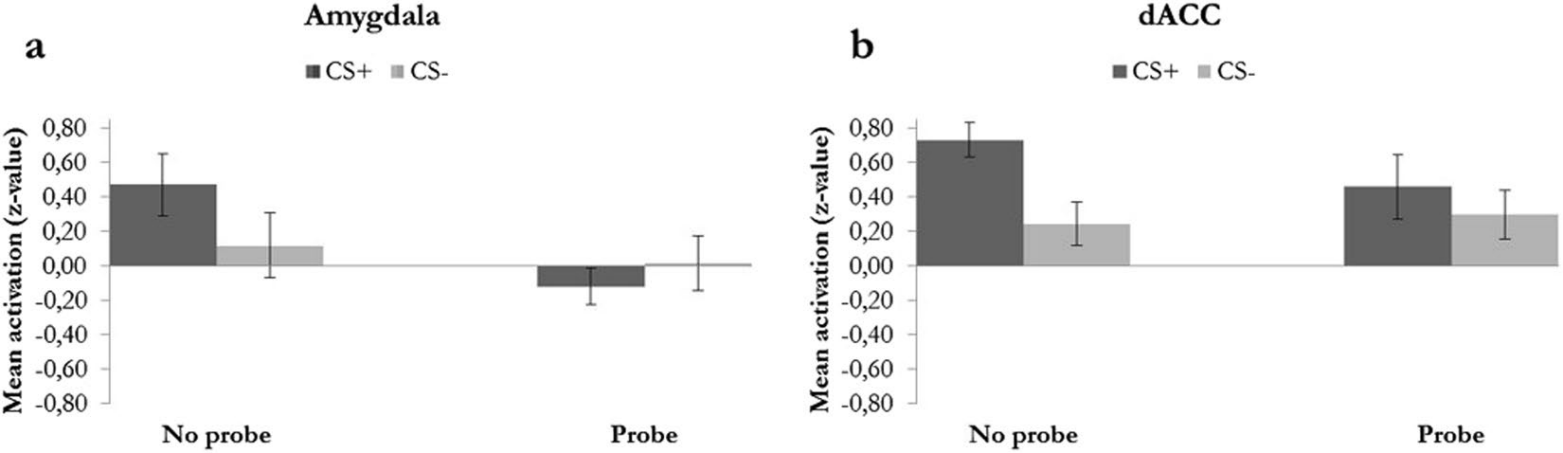
(**a**) Mean z-values for the amygdala and for the dACC (**b**) for CS+_Probe_, CS−_Probe_, CS+_No probe_ and CS−_No probe_ extracted from single subject z-stats. Error bars represent standard error of the mean.

Subsequently, we performed additional ROI analyses to obtain an estimation of the non-biased effect sizes. We extracted mean z-values from single subject z-stats from the individual ROIs for the contrasts CS+_Probe_, CS−_Probe_, CS+_No probe_ and CS−_No probe_ and performed a repeated measures ANOVA (Stimulus × Condition) (Table 3). We found a significant main effect of Stimulus in the dACC (*F(*1,17) = 10.33, *p* = 0.05, η_p_^2^ = 0.378), insula *F(*1,17) = 6.19, *p* = 0.024, η_p_^2^ = 0.267 uncorrected) and ventral striatum *F(*1,17) = 26.60, *p* < 0.01, η_p_^2^ = 0.610). We neither observed a significant main effect of Condition (η_p_^2^ range from <0.01 in midbrain to 0.19 in amygdala) nor a significant interaction between Stimulus and Condition; the effect sizes (η_p_^2^) ranged from 0.018 in the insula to 0.143 in amygdala and 0.147 in dACC. For exploration of the direction of the effect in the amygdala (resulting from the voxel wise analysis described above), and in the dACC (not significant, but showing the largest effect size in the ROI analysis) the mean z-values per condition are depicted in Fig. 5a and b. Further, to make sure that we do not falsely dismiss a possible effect of acoustic startle probes on fear learning (as measured with fMRI) due to a lack of power, we performed sample size calculations to estimate the sample size that would be required to be able to observe a significant interaction effect (G*Power version 3.1.9.2 ANOVA: Repeated measures, within factors with correlation among repeated measures: 0.5). We would have needed a sample size of 4208 for the insula (η_p_^2^ = 0.018) and a sample size of 65 for dACC (η_p_^2^ = 0.147) to obtain significant interactions with 80% power at 5% alpha.

**Table 3:** Stimulus (CS+/CS−) × Condition (Probe/No probe) repeated measures ANOVA on single subject mean z-stats extracted from the individual ROIs for the contrasts CS+_Probe_, CS−_Probe_, CS+_No probe_ and CS−_No probe_.

	Main effect of Stimulus (2)		Main effect of Condition (2)		Interaction Stimulus(2) × Condition (2)	
	F		F		F	
	*p*-value	η_p_^2^	*p*-value	η_p_^2^	*p*-value	η_p_^2^
dACC	10.332		0.599		2.920	
	0.005*	0.378	*0.450*	0.034	*0.106*	0.147
Insula	6.188		0.370		0.313	
	*0.024*	0.267	*0.551*	0.021	*0.583*	0.018
Thalamus	1.523		0.043		1.836	
	*0.234*	0.082	*0.838*	0.003	*0.193*	0.097
Ventral striatum	26.604		0.124		1.464	
	<0.001*	0.610	*0.729*	0.007	*0.243*	0.079
Amygdala	0.882		3.909		2.847	
	*0.361*	0.049	*0.064*	0.187	*0.110*	0.143
Midbrain	1.160		0.000		0.928	
	*0.297*	0.064	*0.992*	<0.001	*0.349*	0.052

*Significant after Bonferroni correction of α for the number of ROIs (six ROIs).

## Discussion

Successful differential fear conditioning was observed on both the physiological and the behavioral measures of fear. In line with previous studies, pupil (No probe condition) and US-expectancy ratings (Probe and No probe condition) showed conditioned responding to the CS+ compared to the CS−^15,22,23,30-32^. Also, increased activity to the CS+ compared to the CS− was observed in the dACC, insula and ventral striatum. This activation of regions in the fear-network is consistent with previous fMRI studies on fear conditioning^21^. Moreover, previous studies have implicated ACC and insula activation in fear learning and the expression of the fear response mainly after knowledge of the CS−US contingency has been established^21,46,47^ and activation of ventral striatum has been found to play a role in fear learning and in the prediction of salient cues in general^21,46,48^. In addition to the overall effect of fear conditioning, we found that acoustic startle probes influenced pupil dilation by increasing pupil responses to the CS−, and we found a trend significant interaction between Stimulus and Condition in US-expectancy ratings and in brain activation in the amygdala. Both the interaction between Stimulus and Condition in pupil dilation (Fig. 3) and in US-expectancy ratings (Fig. 2) showed a pattern of decreased differentiation between CS+ and CS−, caused by an increased responding to CS− in the probe condition (not tested for US-expectancy). Note however that retrospective US-expectancy ratings were used and that this might be less sensitive than online US-expectancy ratings. The increased pupil response to CS− in the Probe condition, together with the finding that participants reported to perceive the startle probe as equally aversive and arousing as the US, suggests that the startle probe elicits an anticipatory pupil dilation response similar to the US. This suggests that arousal, and thereby orienting^33,34^, might be increased by the acoustic startle probe. The CS− in the Probe condition might therefore be perceived as less safe than the CS− in the No probe condition, reducing safety learning. Furthermore, acoustic startle probes were administered on every Probe trial, but a task with a partial scheme (probe administered on a percentage of the trials) is likely to show the same effect because the anticipation of the acoustic startle probe (similar to US anticipation) causes the increase in pupil dilation for the CS−. These findings are not in line with previous results by Sjouwerman and colleagues showing that acoustic startle probes decreased skin conductance responding for CS+^20^. A possible explanation of the discrepancy may be that Sjouwerman and colleagues used skin conductance instead of pupil dilation and a between subject design, requiring a different interpretation of response amplitudes than in our within-subject design. Another difference between studies is the 100% US reinforcement rate (vs 46% in the current study) which could potentially lead to a habituation of the negative valence of the CS+ thereby attenuating the startle fear response. We did not observe an effect of acoustic startle probes on midbrain activity, even though pupil dilation is thought to be a direct measure of LC activation^26^. We might have been able to detect a possible effect of acoustic startle probes on LC activity, if the spatial resolution and scan parameters had been optimized for LC imaging. In addition, we did not find a significant (interaction) effect of probes in the dACC, insula and thalamus, regions in which activation seems to correlate with pupil dilation during fear learning^15^. However, a (relatively) large effect size for the interaction between Stimulus and Condition (0.147) was found in the dACC, the region that correlates strongest with pupil dilation^15^. Likewise, we observed a significant interaction effect that did not survive Bonferroni correction (for testing multiple ROIs) in the amygdala. We did not perform post-hoc tests to further assess the potential effect. Together, these findings suggest that acoustic startle probes affect fear learning to some extent and that this effect is likely to be mediated through increased arousal. In addition, the finding that acoustic startle probes affect fear learning implies that FPS indeed does not reflect the same fear learning process as pupil dilation.

Importantly, we observed normal fear-network activation and the startle probes did not significantly affect activation in regions of the fear-network, indicated by generally low effect sizes of the Stimulus and Condition interaction.

Unfortunately we were unable to analyze the FPS data because the data acquisition methods of the current study yielded FPS data of insufficient quality. In a previous study our lab was able to measure FPS in the scanner^23^. While we used the same off-line filtering procedures in the current study (and additionally tried alternative ones), the data acquisition set-up was quite different (e.g., different electrodes, different leads, a different amplifier, different software and a lower sampling rate), and resulted in poor-quality EMG data. At present, we do not know what procedural elements are key to obtaining reliable FPS signal in the scanner, but we think that the relatively low sampling rate (1000 Hz) in the current study (cf 2048 in van Well *et al*., 2013) is likely to be the main problem. The very limited number of published studies combining acoustic FPS and fMRI suggests that this is still very challenging^22,23^. Specifically combining FPS and fMRI seems to be a problem, since other forms of EMG measurements, with signals of interest in a different frequency range than FPS, have been successfully combined with fMRI^49–51^.

A limitation of the current study is the relatively large number of participants that had to be excluded from analysis (n = 10). Some of them had to be excluded because of technical difficulties with the setup, but the majority had to be excluded because of sleepiness. This was surprising considering that both very loud noises and electrical shocks were administered during our task, but the relatively long inter-trial-intervals (M = 15 s) might nevertheless have caused participants to dose off. In addition, several parameters might influence accurate measurement of pupil dilation: whether or not a fixation cross is presented on top of the pictures, the visual angle of the pictures and whether gaze shift related artefacts differ between conditions. We cannot completely rule out that our data is somehow influenced by these parameters. To summarize, acoustic startle probes influence fear learning by enhancing the pupil dilation to the safety cue. A similar effect of probes on retrospective US-expectancy ratings (trend significant) was observed and, in addition, acoustic startle probes may also affect fear learning in the amygdala(trend significant).

In conclusion, acoustic startle probes affect differential fear learning by impeding safety learning, as measured with pupil dilation, a read-out of the cognitive component of fear learning. However, we observed no significant effect of startle probes on other measures of fear learning (retrospective US-expectancy ratings and BOLD fMRI of the fear-network). Hence, a possible effect of startle probes on cognitive read-outs of fear learning should be taken into consideration when deciding on the preferred measures of interest, but we found no further evidence against combining multiple read-outs of fear-learning within one experiment.

## References

[1] Baeyens F, Eelen P, Crombez G (1995). Pavlovian associations are forever: On classical conditioning and extinction. Journal of Psychophysiology.

[2] Purkis HM, Lipp OV (2001). Does affective learning exist in the absence of contingency awareness?. Learn Motiv.

[3] LaBar KS, Cabeza R (2006). Cognitive neuroscience of emotional memory. Nat Rev Neurosci.

[4] Hamm AO, Vaitl D (1996). Affective learning: awareness and aversion. Psychophysiology.

[5] Hamm AO, Weike AI (2005). The neuropsychology of fear learning and fear regulation. Int J Psychophysiol.

[6] Squire LR (2004). Memory systems of the brain: a brief history and current perspective. Neurobiol Learn Mem.

[7] Sevenster D, Beckers T, Kindt M (2012). Instructed extinction differentially affects the emotional and cognitive expression of associative fear memory. Psychophysiology.

[8] Soeter M, Kindt M (2010). Dissociating response systems: erasing fear from memory. Neurobiol Learn Mem.

[9] Lonsdorf TB (2017). Don’t fear ‘fear conditioning’: Methodological considerations for the design and analysis of studies on human fear acquisition, extinction, and return of fear. Neurosci Biobehav Rev.

[10] Davis M (2006). Neural systems involved in fear and anxiety measured with fear-potentiated startle. Am Psychol.

[11] Bos MG, Jentgens P, Beckers T, Kindt M (2013). Psychophysiological response patterns to affective film stimuli. PLoS One.

[12] Bradley MM, Miccoli L, Escrig MA, Lang PJ (2008). The pupil as a measure of emotional arousal and autonomic activation. Psychophysiology.

[13] Sevenster D, Beckers T, Kindt M (2014). Fear conditioning of SCR but not the startle reflex requires conscious discrimination of threat and safety. Front Behav Neurosci.

[14] Soeter M, Kindt M (2011). Disrupting reconsolidation: pharmacological and behavioral manipulations. Learn Mem.

[15] Leuchs L, Schneider M, Czisch M, Spoormaker VI (2016). Neural correlates of pupil dilation during human fear learning. Neuroimage.

[16] Weike AI (2005). Fear conditioning following unilateral temporal lobectomy: dissociation of conditioned startle potentiation and autonomic learning. J Neurosci.

[17] Dichter GS, Tomarken AJ, Baucom BR (2002). Startle modulation before, during and after exposure to emotional stimuli. Int J Psychophysiol.

[18] Mallan KM, Lipp OV, Libera M (2008). Affect, attention, or anticipatory arousal? Human blink startle modulation in forward and backward affective conditioning. Int J Psychophysiol.

[19] Lissek S (2005). Airpuff startle probes: an efficacious and less aversive alternative to white-noise. Biol Psychol.

[20] Sjouwerman R, Niehaus J, Kuhn M, Lonsdorf TB (2016). Don’t startle me-Interference of startle probe presentations and intermittent ratings with fear acquisition. Psychophysiology.

[21] Fullana MA (2016). Neural signatures of human fear conditioning: an updated and extended meta-analysis of fMRI studies. Mol Psychiatry.

[22] Lindner K (2015). Fear-potentiated startle processing in humans: Parallel fMRI and orbicularis EMG assessment during cue conditioning and extinction. Int J Psychophysiol.

[23] van Well S, Visser RM, Scholte HS, Kindt M (2012). Neural substrates of individual differences in human fear learning: evidence from concurrent fMRI, fear-potentiated startle, and US-expectancy data. Cogn Affect Behav Neurosci.

[24] Bach DR, Flandin G, Friston KJ, Dolan RJ (2009). Time-series analysis for rapid event-related skin conductance responses. J Neurosci Methods.

[25] Benedek M, Kaernbach C (2010). A continuous measure of phasic electrodermal activity. J Neurosci Methods.

[26] Koss MC (1986). Pupillary dilation as an index of central nervous system alpha 2-adrenoceptor activation. J Pharmacol Methods.

[27] Aston-Jones G, Bloom FE (1981). Norepinephrine-containing locus coeruleus neurons in behaving rats exhibit pronounced responses to non-noxious environmental stimuli. J Neurosci.

[28] Wang CA, Boehnke SE, Itti L, Munoz DP (2014). Transient pupil response is modulated by contrast-based saliency. J Neurosci.

[29] Reinhard G, Lachnit H, Konig S (2006). Tracking stimulus processing in Pavlovian pupillary conditioning. Psychophysiology.

[30] Visser RM (2016). Quantifying learning-dependent changes in the brain: Single-trial multivoxel pattern analysis requires slow event-related fMRI. Psychophysiology.

[31] Visser RM, Kunze AE, Westhoff B, Scholte HS, Kindt M (2015). Representational similarity analysis offers a preview of the noradrenergic modulation of long-term fear memory at the time of encoding. Psychoneuroendocrinology.

[32] Visser RM, Scholte HS, Beemsterboer T, Kindt M (2013). Neural pattern similarity predicts long-term fear memory. Nat Neurosci.

[33] Sara SJ, Bouret S (2012). Orienting and reorienting: the locus coeruleus mediates cognition through arousal. Neuron.

[34] Wang CA, Munoz DP (2015). A circuit for pupil orienting responses: implications for cognitive modulation of pupil size. Curr Opin Neurobiol.

[35] Henckens MJ, Hermans EJ, Pu Z, Joels M, Fernandez G (2009). Stressed memories: how acute stress affects memory formation in humans. J Neurosci.

[36] Coste CP, Kleinschmidt A (2016). Cingulo-opercular network activity maintains alertness. Neuroimage.

[37] Sadaghiani S, D’Esposito M (2015). Functional Characterization of the Cingulo-Opercular Network in the Maintenance of Tonic Alertness. Cereb Cortex.

[38] Blumenthal TD (2005). Committee report: Guidelines for human startle eyeblink electromyographic studies. Psychophysiology.

[39] Lemieux L, Allen PJ, Franconi F, Symms MR, Fish DR (1997). Recording of EEG during fMRI experiments: patient safety. Magn Reson Med.

[40] Bradley MM, Lang PJ (1994). Measuring emotion: the Self-Assessment Manikin and the Semantic Differential. J Behav Ther Exp Psychiatry.

[41] Jenkinson M, Bannister P, Brady M, Smith S (2002). Improved optimization for the robust and accurate linear registration and motion correction of brain images. Neuroimage.

[42] Smith SM (2002). Fast robust automated brain extraction. Hum Brain Mapp.

[43] Smith SM, Nichols TE (2009). Threshold-free cluster enhancement: addressing problems of smoothing, threshold dependence and localisation in cluster inference. Neuroimage.

[44] Ezra M, Faull OK, Jbabdi S, Pattinson KT (2015). Connectivity-based segmentation of the periaqueductal gray matter in human with brainstem optimized diffusion MRI. Hum Brain Mapp.

[45] Keren NI, Lozar CT, Harris KC, Morgan PS, Eckert MA (2009). *In vivo* mapping of the human locus coeruleus. Neuroimage.

[46] Greco JA, Liberzon I (2016). Neuroimaging of Fear-Associated Learning. Neuropsychopharmacology.

[47] Maier S (2012). Clarifying the role of the rostral dmPFC/dACC in fear/anxiety: learning, appraisal or expression?. PLoS One.

[48] Pohlack ST, Nees F, Ruttorf M, Schad LR, Flor H (2012). Activation of the ventral striatum during aversive contextual conditioning in humans. Biol Psychol.

[49] Contarino MF (2012). Is there a role for combined EMG-fMRI in exploring the pathophysiology of essential tremor and improving functional neurosurgery?. PLoS One.

[50] Dietz V (2015). Neural coupling of cooperative hand movements: a reflex and fMRI study. Cereb Cortex.

[51] Neuner I (2010). Electrophysiology meets fMRI: neural correlates of the startle reflex assessed by simultaneous EMG-fMRI data acquisition. Hum Brain Mapp.

